# Transcriptome sequencing and analysis of the zoonotic parasite *Spirometra erinacei* spargana (plerocercoids)

**DOI:** 10.1186/1756-3305-7-368

**Published:** 2014-08-15

**Authors:** Dae-Won Kim, Won Gi Yoo, Myoung-Ro Lee, Hye-Won Yang, Yu-Jung Kim, Shin-Hyeong Cho, Won-Ja Lee, Jung-Won Ju

**Affiliations:** Division of Malaria and Parasitic Diseases, Centre for Immunology and Pathology, Korea National Institute of Health, Chungbuk, 363-951 Republic of Korea; Department of Parasitology, Kyungpook National University School of Medicine, Daegu, 702-701 Republic of Korea

**Keywords:** *Spirometra erinacei*, Sparganum, Plerocercoids, Transcriptome, Sequencing

## Abstract

**Background:**

Although spargana, which are the plerocercoids of *Spirometra erinacei*, are of biological and clinical importance, expressed sequence tags (ESTs) from this parasite have not been explored. To understand molecular and biological features of this parasite, sparganum ESTs were examined by large-scale EST sequencing and multiple bioinformatics tools.

**Methods:**

Total RNA was isolated from spargana and then ESTs were generated, assembled and sequenced. Many biological aspects of spargana were investigated using multi-step bioinformatics tools.

**Results:**

A total of 5,634 ESTs were collected from spargana. After clustering and assembly, the functions of 1,794 Sparganum Assembled ESTs (SpAEs) including 934 contigs and 860 singletons were analyzed. A total of 1,351 (75%) SpAEs were annotated using a hybrid of BLASTX and InterProScan. Of these genes, 1,041 (58%) SpAEs had high similarity to tapeworms. In the context of the biology of sparganum, our analyses reveal: (i) a highly expressed fibronectin 1, a ubiquitous and abundant glycoprotein; (ii) up-regulation of enzymes related with glycolysis pathway; (iii) most frequent domains of protein kinase and RNA recognition motif domain; (iv) a set of helminth-parasitic and spargana-specific genes that may offer a number of antigen candidates.

**Conclusions:**

Our transcriptomic analysis of *S. erinacei* spargana demonstrates biological aspects of a parasite that invades and travels through subcutaneous tissue in intermediate hosts. Future studies should include comparative analyses using combinations of transcriptome and proteome data collected from the entire life cycle of *S. erinacei*.

**Electronic supplementary material:**

The online version of this article (doi:10.1186/1756-3305-7-368) contains supplementary material, which is available to authorized users.

## Background

Spargana, the plerocercoid form of *Spirometra erinacei,* are the larvae of intestinal tapeworms of the order Diphyllobothriidea in the class Cestoda [1]. Sparganosis has been reported in many countries, including the United States and Europe [2]. Human sparganosis occasionally occurs by ingestion of water contaminated with Copepods that have been infected with procercoids or by invasion of plerocercoids from hosts such as frogs and snakes.

The ingested sparganum has the ability to invade various organs, which include eyes, subcutaneous tissues, abdominal walls, brains, spinal cords, lungs, and breasts, among others [3–5]. Human sparganosis can cause diverse symptoms, such as non-specific irritation, uncertain pain, apparent masses, and headaches. Although radiologic examinations have been introduced, using techniques such as ultrasonography, CT, and MR, it is difficult to confirm a correct diagnosis. Because expensive equipment and experts are necessary, this approach is not appropriate as a practical method for field diagnosis. Furthermore, sparganosis cannot even be deciphered by autopsy because of restrictions, which include many latent infections, unexpected locations of the worm in the body and a low predicted infection rate [6].

Serodiagnostic tests using sparganum antigen proteins could be good alternative techniques for diagnosing sparganosis. These tests include enzyme-linked immunosorbent assays (ELISA) [7] and immunoblotting [8]. Several antigenic proteases are reportedly present in spargana, including 31/36 kDa excretory-secretory (ES) proteins [9], a 27 kDa cathepsin S-like protease [10], and a 53 kDa neutral protease [11]. ES proteins in crude extracts have been shown to be highly specific and sensitive in sera from patients with sparganosis. However, preparation of sufficient amounts of ES proteins is labor-intensive and time-consuming [12]. Therefore, recombinant antigens were employed to overcome the disadvantages of ES protein preparation. Recently, multiple antigen mixtures using combinations of these antigenic proteins have been recommended because an absolute antigen with high sensitivity and specificity does not yet exist [13].

As mentioned above, the first definitive treatment is surgical resection of the worm from the infected tissues. The second choice for treating sparganosis is two drugs, praziquantel or mebendazole, which are also recommended for treatment of trematode or nematode infections, respectively [14, 15]. Although these drugs are currently orally administrated for treatment, low cure rates and high recurrence rates have already been observed [16, 17]. Because novel therapeutic targets against sparganosis are not studied, with the exception of these drugs, development of anti-helminthics should be actively encouraged.

Large-scale sequencing data can be applied to gene-based discovery of drug targets and diagnostic antigens [18]. Recently, genomes or transcriptomes from other cestode parasites have been sequenced and functionally analyzed, including data from *Taenia solium*[19–21], *Echinococcus multilocularis*[21]*, E. granulosus*[21, 22] and *Hymenolepis microstoma*[21]. This genetic information has been applied to understanding a number of metabolic mechanisms used for parasite growth and during host-parasite interactions. Furthermore, monitoring fluctuations in gene expression is indispensable for finding drug targets, predicting secretory proteins, and elucidating evolutionary relationships [18, 21, 23]. Currently, however, knowledge regarding the genome or transcriptome of various developmental stages in *S. erinacei* is restricted to adult worms.

In this study, a major expressed sequence tags (ESTs) sequencing project on *S. erinacei* spargana was carried out to improve a basic genetic resource. This transcriptome profile is presented with the abundant transcripts, frequently occurring functional domains and antigen candidates.

## Methods

### Sample collection

Spargana of *S. erinacei* were collected from naturally infected *Rhabdophis tigrinus* snakes in Gyeong-sangnam-do province, South Korea. All worms were washed with physiological saline several times and either used directly for RNA preparation or stored at -70°C until use.

### RNA isolation and cDNA library construction

After separating the mycelia from *S. erinacei* spargana, the worms were submerged in liquid nitrogen in pre-chilled grinding jars and a grinding ball on a bed of dry ice. Spargana in pre-chilled grinding jars were pulverized using a Mixer Mill MM301 (Retsch GmbH, Germany). Spargana were transferred to 15 ml polypropylene tubes filled with liquid nitrogen and stored at -80°C. Total RNA was extracted from the fragmented frozen tissues using TRI reagent (MRCgene, OH, USA). Total RNA was purified (100 μg) using the absolutely mRNA Purification Kit (Stratagene, CA, USA) according to the manufacturer’s instructions. To construct the cDNA library, a directional λ ZAP cDNA synthesis/Gigapack III gold cloning kit (Stratagene, CA, USA) was used. Reverse transcription of mRNA for first stand cDNA synthesis was primed from the poly-A tail using an oligo-dT linker primer containing an *Xho*I cloning site. Following second strand synthesis, *EcoR*I linkers were ligated to the 5′-termini. *Xho*I digestion released the *Eco*RI adapter and residual linker primer from the 3′ end of the cDNA. These two fragments were separated on a drip column containing Sepharose® CL-2B gel filtration medium. The fractionated cDNA (above 500 bp) was then precipitated and ligated into the ZAP Express vector (pBK-CMV). The primary library was produced by *in vitro* packaging of the ligation product with a ZAP Express cDNA Gigapack III Gold cloning Kit.

### cDNA sequencing

cDNA clones were plated onto LB-kanamycin plates (Rectangle, 23.5 cm × 23.5 cm) with X-gal and IPTG for blue/white selection. White colonies were randomly and manually picked, inoculated into 15 384-well plates (Corning, NY, USA) containing 40 μl TB/kanamycin and incubated for 16 h at 37°C with fixation culture. Sequences of the cDNA inserts were determined from the 5′ end of clones using the BigDye Terminator Sequencing Kit ver. 3.1 (Applied Biosystems, Foster City, CA, USA) and a 3730XL DNA analyzer (Applied Biosystems).

### EST cleaning and clustering

**Figure 1:**
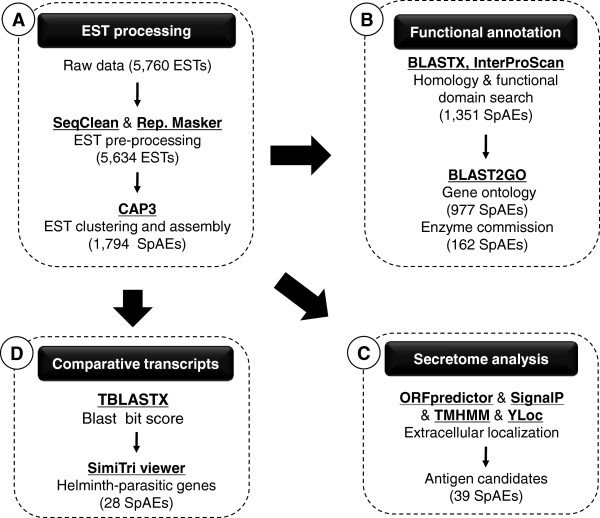
**Main workflow for analysis.** Outlay of analysis steps performed for *Spirometra erinacei* ESTs data. External programs used for analysis are shown where appropriate. ESTs were pre-processed and subjected to clustering and assembly **(A)**. Singlets and contigs were examined for homology **(B)**, screened for secretory antigen candidates **(C)** and compared with other species at the whole transcriptome scale **(D)**.

The ESTs were initially analyzed and annotated using PESTAS, an automated EST analysis platform [24]. In our study, the analysis pipeline consisted of three steps (Figure 1). In step I, EST trace data from *S. erinacei* sparganum were base-called from trace chromatogram data using Phred quality scores of 13 [25, 26]. The sequences were then processed with Cross_Match (http://www.phrap.org), RepeatMasker (http://www.repeatmasker.org/) and SeqClean (http://seqclean.sourceforge.net/) to filter out sequences from vectors, *E. coli*, repetitive elements and mitochondrial DNA. Trimmed sequences over 100 bp in length were clustered and assembled into putative unique EST objects by TGICL [27] and CAP3 [28], using the default options.

### Homology search and functional annotation

To assign putative functions to *S. erinacei* ESTs, we took into account the BLASTX best hit descriptions and subsequent alignments with E-value cutoffs below 1e-10 and compared them to the non-redundant (NR) protein database at NCBI. Because a large portion of these ESTs have not yet been annotated, we further characterized domains/families of the SpAEs using InterPro database version 27 (HMMPfam, HMMSmart, HMMTigr, HMMPanther and SuperFamily; flagged as true by InterProScan with E-value ≤ 1e-4) [29]. We also classified our SpAEs with Gene Ontology (GO) terms at the protein level using BLAST2GO (cut-off E-value ≤ 1e-10) [30]. These GO terms were further mapped and classified at the third level to two GO categories: ‘molecular function,’ and ‘biological process.’ Because some predicted proteins were assigned to more than one GO term, the percentages of each category add up to one hundred percent. SpAEs also were mapped to the Enzyme Commission (EC) database via BLAST2GO.

### Comparative transcriptome analysis

Gene sequences of spargana were globally compared to those of other species using TBLASTX (E-value 1e-5) and the results were displayed using the SimiTri program (BLAST score cut-off score: 50) [31]. Sequences of the comparator species were downloaded from the GenBank databases.

### Secretome analysis

From the ORFs inferred from SpAEs, secreted proteins were predicted using a combination of four programs (ORFpredictor [32], SignalP [33], TMHMM [33] and YLoc [34]) to minimize the number of false positive predictions. Firstly, we identified protein-coding regions of ORFs in SpAEs by starting exactly at the initiation codon encoding the amino acid methionine (Met) with ORFpredictor. Secondly, SignalP 3.0 was used to predict the presence of secretory signal peptides and signal anchors for each predicted SpAE protein, using both neural networks and Hidden Markov models with default option. To exclude erroneous predictions of putative transmembrane (TM) sequences as signal sequences, TMHMM, a membrane topology prediction program, was applied. We further validated the list of secreted proteins with extracellular localization using YLoc.

## Results and discussion

### Overview of sparganum EST analysis

**Figure 2:**
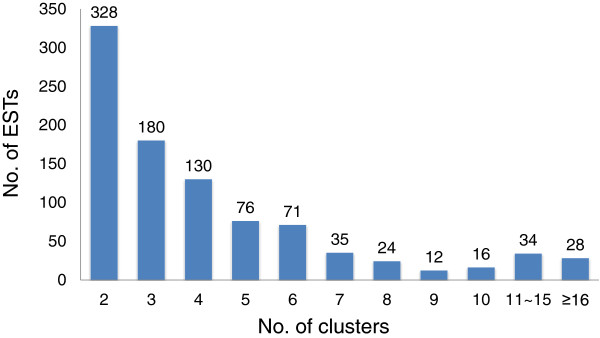
**Distribution of ESTs within contigs after clustering the 5,634 sequences using CAP3.**

Of the 5,760 clones sequenced, a total of 5,634 high-quality ESTs (an average read length of 687 bp) were obtained with a 97.8% sequencing success rate, after trimming vector contamination and low quality bases and eliminating trimmed sequences less than 100 bp in length. A total of 1,794 SpAEs (Sparganum Assembled ESTs, average read length of 715 bp) were obtained after clustering a set of 5,634 ESTs (Figure 1A). The set of SpAEs is comprised of 934 contigs and 860 singletons (Table 1). Average sequence lengths for the contigs and singletons were 764 bp and 661 bp, respectively. The contigs were mostly composed of two to six ESTs (Figure 2), with a maximum of 164 different ESTs in a single contig (Additional file 1: Table S1). All trimmed ESTs were deposited into the NCBI GenBank with continuous accession numbers of HS514072-HS519705.

**Table 1:** **Transcriptome features of** ***S. erinacei*** **spargana**

	Numbers
Total sequence reads	5,760
Total analyzed reads (average size)	5,634 (687 bp)
Total number of assembled sequences (average size)	1,794 (715 bp)
Contigs	934
Singlets	860
Total annotated genes (BLASTX or InterProScan)	1,351
BLASTX	1,335
InterProScan	96

### Functional annotation of SpAEs

To identify likely *S. erinacei* sparganum genes through sequence similarity, BLASTX analyses and InterProScan domain searches were performed on all SpAEs against the NCBI NR protein databases and the InterProScan database (Figure 1B). The two alignment algorithms were used to annotate 1,351 SpAEs (75%), and most matches were to tapeworms, such as *E. granulosus* and *H. microstoma* (Additional file 2: Figure S1). After removing redundant protein hits, 1,335 unique reference proteins were identified within public databases. Among them, 1,268 (95%) of the annotated SpAEs had E-values of ≤ 1e-10 (Additional file 1: Table S1). In our study, 443 SpAEs (30%) did not share sequence similarity with any other predicted or known molecules in public databases. These SpAEs potentially represent novel genes with unknown functions in *S. erinacei* spargana.

#### Gene ontology

Annotation of EST-derived sparganum genes was implemented on the basis of existing annotation available in public databases. These annotations followed gene ontology (GO) vocabularies for organization into two categories representing biological processes and molecular functions [35]. In our study, 977 of the total 1,794 SpAEs could be assigned to biological process (BP) and molecular function (MF) GO classifications through BLAST2GO [30]. All of the SpAEs defined in the GO database could be assigned to more than one ontology. Of the 977 SpAEs mapped with GO terms below level 3, 669 SpAEs had BP annotation and 825 SpAEs had MF annotation. Among genes annotated with BPs, the most highly scored categories were Cellular macromolecule metabolic process (GO:0044260, 31.83%), Cellular protein metabolic process (GO:0044267, 24.51%), Gene expression (GO:0010467, 19.13%) and Translation (GO:0006412, 12.25%). The largest proportion of MFs for the SpAEs were involved in ATP binding (GO:0005524, 12.12%), Purine ribonucleoside binding (GO:0032550, 16.36%), Purine ribonucleoside triphosphate binding (GO:0035639, 16.36%) and Nucleoside phosphate binding (GO:1901265, 22.54%) (Table 2). Spargana grow into their adult stages in the final host. To achieve this developmental transition, various proteins, such as structural proteins or metabolic proteins, should be produced through translation. Both BP and MF exhibited high ranked GO categories that elucidated physiological features of spargana, including protein synthesis, protein transport, and protein regulation.

**Table 2:** **Biological process and molecular function GO terms with the 15 highest scores**

Category	Level	GO ID	GO terms	Representation ^a^		Score ^c^
				Number	Percentage ^b^	
Biological process	5	GO:0044260	Cellular macromolecule metabolic process	213	31.84%	74.85
	6	GO:0044267	Cellular protein metabolic process	164	24.51%	73.52
	5	GO:0010467	Gene expression	128	19.13%	71.09
	7	GO:0006412	Translation	82	12.26%	70.64
	6	GO:0034645	Cellular macromolecule biosynthetic process	123	18.39%	62.53
	4	GO:0043170	Macromolecule metabolic process	229	34.23%	62.04
	5	GO:0019538	Protein metabolic process	179	26.76%	55.76
	4	GO:0050794	Regulation of cellular process	107	15.99%	45.18
	4	GO:0044249	Cellular biosynthetic process	146	21.82%	42.06
	5	GO:0009059	Macromolecule biosynthetic process	123	18.39%	37.73
	6	GO:0016310	Phosphorylation	53	7.92%	34.29
	4	GO:0006810	Transport	76	11.36%	29.17
	8	GO:0006468	Protein phosphorylation	35	5.23%	28.24
	5	GO:0007165	Signal transduction	58	8.67%	28.05
	4	GO:0055114	Oxidation-reduction process	42	6.28%	27.96
Molecular function	9	GO:0005524	ATP binding	100	12.12%	100
	6	GO:0032550	Purine ribonucleoside binding	135	16.36%	84
	5	GO:0035639	Purine ribonucleoside triphosphate binding	135	16.36%	82.2
	4	GO:1901265	Nucleoside phosphate binding	186	22.55%	78.48
	4	GO:0043168	Anion binding	162	19.64%	70.46
	4	GO:0003676	Nucleic acid binding	112	13.58%	69.51
	5	GO:0000166	Nucleotide binding	186	22.55%	66.35
	8	GO:0032559	Adenyl ribonucleotide binding	100	12.12%	61.8
	5	GO:0046872	Metal ion binding	88	10.67%	54.83
	5	GO:0032549	Ribonucleoside binding	136	16.48%	52.84
	5	GO:0001883	Purine nucleoside binding	135	16.36%	50.4
	7	GO:0032555	Purine ribonucleotide binding	135	16.36%	50.4
	5	GO:0003723	RNA binding	56	6.79%	38.19
	7	GO:0030554	Adenyl nucleotide binding	100	12.12%	37.8
	9	GO:0005525	GTP binding	37	4.48%	37

^a^Note that individual GO categories can have multiples mappings. The representation means the number of SpAEs that can be mapped to a certain GO term.

^b^The representation percentage is based on the total number of GO mappings in each of the two major ontologies (biological process: 669, molecular function: 825).

^c^Score was calculated by BLAST2GO according to number of different sequences annotated at a child GO term and distance to node of the child GO term.

#### Highly abundant genes

We determined, as highly abundant genes, SpAEs with more than fourteen ESTs in one contig after exclusion of ribosomal RNA and mitochondrial genes (Table 3). In an attempt to characterize highly expressed genes, there were active components in the metabolism of the parasite, including fructose-bisphosphate aldolase (FBA) and glyceraldehyde-3-phosphate dehydrogenase. Their up-regulation may be required for high metabolic activity during development [18]. Plerocercoid growth factor/cysteine protease and signal peptidase complex subunit 3 also were found, of which cysteine proteinase has been previously investigated for their role in parasite-host relationship [36]. In our study, fibronectin 1 (FN1), which was represented by 164 ESTs, was the most frequently expressed gene. FN is a ubiquitous and abundant glycoprotein. FN consists of three discrete domains composed of FN1, FN2, and FN3. Interaction of FN with different receptors is important for mediating cellular adhesion and migration processes such as embryonic development and wound healing [37]. FN can also modulate host defenses by binding to immunoglobulin molecules like IgG and immobilizing them on a solid matrix [38]. Although FN functions are poorly studied in parasites, it is speculated that FN provides a structural basis for cell adhesion, transduces signals for cell proliferation and apoptosis, and serves for defenses against the host [38, 39].

**Table 3:** **The most abundant transcripts in** ***S. erinacei*** **spargana**

Cluster ID	No. of reads	Accession ID	Description	Organism	E-value
EPA018LGAA12C000033	164	XP_007424327.1	PREDICTED: fibronectin isoform X1	*Python bivittatus*	1.73E-91
EPA018LGAA12C000039	90	EUB60510.1	Polyadenylate-binding protein	*Echinococcus granulosus*	0
EPA018LGAA12C000019	87	AAD11479.1	Cytoplasmic antigen containing repeat epitope, partial	*Spirometra erinaceieuropaei*	0
EPA018LGAA12C000001	80	EUB65008.1	Cyclin-I	*Echinococcus granulosus*	2.45E-69
EPA018LGAA12C000005	70	AFX72984.1	Elongation factor 1 alpha	*Spirometra erinaceieuropaei*	0
EPA018LGAA12C000052	61	-	-		-
EPA018LGAA12C000025	50	AAL18701.1	AF418991_1 cytoplasmic antigen 4	*Spirometra erinaceieuropaei*	1.88E-62
EPA018LGAA12C000035	38	GAA43229.2	ATP-dependent RNA helicase UAP56/SUB2	*Clonorchis sinensis*	1.6E-132
EPA018LGAA12C000055	38	AFX73009.1	pDJA1 chaperone	*Spirometra erinaceieuropaei*	0
EPA018LGAA12C000053	36	BAA90773.1	Glyceraldehyde-3-phosphate dehydrogenase	*Spirometra erinaceieuropaei*	0
EPA018LGAA12C000018	35	AFM74218.1	40S ribosomal protein S24	*Spirometra erinaceieuropaei*	4.97E-70
EPA018LGAA12C000047	35	CDJ25645.1	Transaldolase	*Echinococcus granulosus*	5.15E-64
EPA018LGAA12C000028	32	CDJ16325.1	Programmed cell death protein 4	*Echinococcus granulosus*	3.03E-88
EPA018LGAA12C000056	27	-	-		-
EPA018LGAA12C000061	26	CAX75788.1	Tubulin beta-2C chain	*Schistosoma japonicum*	0
EPA018LGAA12C000062	24	-	-		-
EPA018LGAA12C000010	22	ABR68549.1	Cystatin-2	*Clonorchis sinensis*	1.49E-19
EPA018LGAA12C000063	21	CDJ08795.1	Nervous system adducin	*Hymenolepis microstoma*	5.3E-113
EPA018LGAA12C000064	21	EUB59337.1	Actin	*Echinococcus granulosus*	0
EPA018LGAA12C000065	21	Q8MUA4.1	14332_ECHGR RecName: Full = 14-3-3 protein homolog 2	*-*	5.4E-101
EPA018LGAA12C000066	21	CDJ25303.1	Synaptic vesicle membrane protein VAT 1	*Echinococcus granulosus*	1.4E-169
EPA018LGAA12C000070	20	BAB62718.1	Plerocercoid growth factor/cysteine protease	*Spirometra erinaceieuropaei*	0
EPA018LGAA12C000009	19	ABN14906.1	Heat shock protein 90 alpha	*Taenia asiatica*	4.31E-71
EPA018LGAA12C000040	19	-	-		-
EPA018LGAA12C000075	18	XP_002020246.1	GL13880	*Drosophila persimilis*	1.1E-107
EPA018LGAA12C000049	16	-	-		-
EPA018LGAA12C000080	16	CDJ23790.1	Heat shock 70 kDa protein 4	*Echinococcus granulosus*	0
EPA018LGAA12C000081	16	CDJ17948.1	gtp binding protein 2	*Echinococcus granulosus*	3.37E-70
EPA018LGAA12C000024	15	CDJ17047.1	40s ribosomal protein s15	*Echinococcus granulosus*	6.88E-65
EPA018LGAA12C000076	15	CDJ20938.1	Ubiquitin conjugating enzyme E2 G1	*Echinococcus granulosus*	8.5E-104
EPA018LGAA12C000082	15	CDJ15210.1	Excitatory amino acid transporter 3	*Hymenolepis microstoma*	2.23E-80
EPA018LGAA12C000085	15	CDJ17337.1	Fructose 16 bisphosphate aldolase	*Echinococcus granulosus*	6.4E-178
EPA018LGAA12C000086	15	CDJ13399.1	Signal peptidase complex subunit 3	*Hymenolepis microstoma*	7E-137

A parasite should adapt to a variety of biological stresses in the host environment, including thermal shock, oxidative stress and other forms of stress [40]. Hence, proteins that allow spargana to survive stresses are important components for infection establishment. We found stress response-related proteins, such as HSP70, HSP40, HSP90, HSP71, HSP105, HSP60 and HSPA8. HSPs are highly conserved and abundant proteins in many parasitic organisms [21, 41, 42] and are essential for cellular viability and activity under both normal and stress conditions [43]. The top 3 most abundant genes are HSP70 (55 reads), HSP40 (47 reads) and HSP90 (24 reads). It has been previously observed that HSP70 and HSP80 in *T. solium* cysticerci were highly induced under temperature stress [44]. Recently, expansion of HSP70 was described in tapeworms and points out the importance of such proteins for the parasite life cycle. HSP40 gets involved in the prevention of protein aggregation and the regulation of protein refolding for parasitic development [45]. HSP90 functions downstream of the HSP70/HSP40-chaperone system and serves as an important determinant in regulating protein conformation and cell signal transduction [46].

#### Abundant domains

A comparison of SpAEs with the Pfam domain database [47] was performed to determine representation of protein families, domains, and functional sites in the sparganum. This analysis revealed matches to 614 unique protein domain families. The Pfam domain families with the most frequent representation in the SpAEs are presented in Table 4. These findings are similar with the result of Parkinson et al. [22], who showed that RNA recognition motif (PF00076), EF-hand domain pair (PF13499) and WD40 repeat (PF00400) were constantly abundant across the Lophotrochozoa. They also reported that dynein light chain (PF01221) and tetraspanin/peripherin (PF00335) appeared expanded in both cestode and trematode. In our study, the most abundant protein motifs were protein kinase domain (PF00069), followed by RNA recognition motif. Protein kinases mediate many other cellular processes including metabolism and transcription and protein kinase domains were consistently abundant in platyhelminthes except for *Echinococcus* species [22, 48]. Additionally, there were various functional domains that were involved in structural, regulatory and developmental activities.

**Table 4:** **The 25 most frequent Pfam domains in** ***S. erinacei*** **spargana**

Protein domain family	Pfam ID	No. of SpAEs
Protein kinase domain	PF00069	22
RNA recognition motif domain	PF00076	20
BTB/Kelch-associated	PF07707	15
EF-hand domain pair	PF13499	13
BTB/POZ	PF00651	12
Chaperonin Cpn60/TCP-1	PF00118	11
Phox/Bem1p	PF00564	10
WD40 repeat	PF00400	10
Small GTPase superfamily	PF00071	9
Heat shock protein 70 family	PF00012	9
Kelch repeat type 1	PF01344	8
Fibronectin, type III	PF00041	8
Null	PF13414	7
Calponin homology domain	PF00307	7
14-3-3 domain	PF00244	7
Ubiquitin-conjugating enzyme, E2	PF00179	7
Thioredoxin domain	PF00085	7
Zinc finger, C2H2	PF13465	6
Leucine rich repeat 4	PF12799	6
Collagen triple helix repeat	PF01391	6
Dynein light chain, type 1/2	PF01221	6
AMP-dependent synthetase/ligase	PF00501	6
Tetraspanin/Peripherin	PF00335	6
Aminotransferase, class V/Cysteine desulfurase	PF00266	6
K Homology domain, type 1	PF00013	6

#### Key enzymes

GO terms derived from the predicted proteins were mapped to Enzyme Commission (EC) numbers. In our study, a total of 162 SpAEs were assigned to 87 unique EC numbers. The top 10 highly represented EC numbers are shown in Table 5. The largest cluster corresponded to 36 ESTs for glyceraldehyde 3-phosphate dehydrogenase (GAPDH), which on the surface of *Trichomonas vaginalis* has been suggested may play a crucial role in providing the parasite with a survival advantage [49]. In addition, we found several enzymes related to glycolysis involving malate dehydrogenase, enolase and FBA. Most parasites utilize glucose and galactose as the main energy sources for a major anaerobic and a minor aerobic respiratory metabolism [50]. Glycolytic enzymes are crucial for the survival and pathogenicity of parasites and thereby have been considered as potential drug targets against protozoan parasites [51–54]. If the parasitic enzymes are highly conserved with human homologs, specificity between parasite and host can be solved using the ability of therapeutic chemistry, combined with new structural features that the enzyme catalytic domains show important parasite-specific structural differences [55, 56] The second-largest cluster was comprised of 35 ESTs for ATP dependent RNA helicase DDX 1 (DEAD box protein 1), which has been identified as essential for parasitic survival [57].

**Table 5:** **The 10 most abundant enzymes in** ***S. erinacei*** **spargana**

Enzyme code	Name	No. of reads	No. of SpAEs	Cluster IDs
EC:1.2.1.12	Glyceraldehyde-3-phosphate dehydrogenase	36	2	EPA018LGAA12C000053, EPA018LGAA12S001658
EC:2.2.1.2	ATP dependent rna helicase ddx1	35	1	EPA018LGAA12C000047
EC:3.4	Cysteine proteinase	20	6	EPA018LGAA12C000070, EPA018LGAA12C000238, EPA018LGAA12C000503, EPA018LGAA12C000561, EPA018LGAA12C000680, EPA018LGAA12S005500
EC:3.6.1.3	Heat shock protein 90 alpha	19	10	EPA018LGAA12C000009, EPA018LGAA12C000086, EPA018LGAA12C000157, EPA018LGAA12C000209, EPA018LGAA12C000367, EPA018LGAA12C000500, EPA018LGAA12C000591, EPA018LGAA12S002094, EPA018LGAA12S004373, EPA018LGAA12S005358
EC:2.6.1.52	Phosphoserine aminotransferase 1	18	1	EPA018LGAA12C000075
EC:4.1.2.13	Fructose-bisphosphate aldolase	15	1	EPA018LGAA12C000085
EC:2.1.1.45	Thymidylate synthase	12	2	EPA018LGAA12C000100, EPA018LGAA12C000380
EC:6.3.1.2	Glutamine synthetase	12	2	EPA018LGAA12C000104, EPA018LGAA12C000121
EC:2.3.1.29	2 amino 3 ketobutyrate coenzyme a ligase	10	1	EPA018LGAA12C000125
EC:1.11.1.7	Glutathione peroxidase	9	1	EPA018LGAA12C000127

### Diagnostic candidate genes based on secretome analysis

ES proteins or other proteins predicted to be expressed on the cell surface have been proposed as diagnostic candidates [58, 59]. Thus, proteins inferred from the sparganum transcriptome were screened for signal peptide and transmembrane domains to find potentially exported proteins. We conducted an analysis of open reading frames (ORFs) containing an N-terminal signal peptide by using multiple bioinformatic tools, such as ORFpredictor, SignalP, TMHMM, and YLoc. A total of 39 SpAEs contained ORFs with extracellular localization sequences (Table 6). The dataset was divided into sequences that were novel and sequences that were found across different phyla. Novel sequences constituted approximately 50% of the total. These genes with no previously identified homologs in other organisms could be particularly intriguing for the development of diagnostic candidates because the lack of host homologs improves the expectation of therapeutic safety and efficacy.

**Table 6:** **Putative secretory proteins predicted by ORFpredictor, SignalP, TMHMM and YLoc**

Cluster ID	No. of reads	Accession ID	E-value	Description	*H. sapiens* (Identity)
EPA018LGAA12C000067	12	-	-	-	-
EPA018LGAA12C000103	12	XP_005104335.1	2.98e-09	PREDICTED: ADP-ribosyl cyclase-like	25%
EPA018LGAA12C000011	10	EUB64644.1	5.25e-08	DNA-binding protein HEXBP	48%
EPA018LGAA12C000266	5	CCD82741.1	2.66e-27	T-cell immunomodulatory protein	27%
EPA018LGAA12C000319	5	ETE62793.1	9.78e-51	Collagen alpha-1(III) chain	66%
EPA018LGAA12C000036	5	AFX72984.1	0	Elongation factor 1 alpha	-
EPA018LGAA12C000355	4	AFI71096.1	1.26e-34	Ag5	31%
EPA018LGAA12C000352	4	-	-	-	-
EPA018LGAA12C000362	4	-	-	-	-
EPA018LGAA12C000007	4	-	-	-	-
EPA018LGAA12C000336	4	-	-	-	-
EPA018LGAA12C000609	3	-	-	-	-
EPA018LGAA12C000487	3	CDJ10900.1	7.93e-50	Phospholipase A	30%
EPA018LGAA12C000593	3	GAA50115.1	3.69e-32	Ribonuclease Oy	33%
EPA018LGAA12C000572	3	CDJ18388.1	4.19e-41	Hypothetical protein EgrG_001045000	34%
EPA018LGAA12C000068	3	-	-	-	-
EPA018LGAA12C000451	3	CDJ13292.1	3.32e-06	Collagen alpha 2(I) chain	53%
EPA018LGAA12C000891	2	XP_003223989.1	6.16e-16	PREDICTED: transforming growth factor-beta-induced protein ig-h3	78%
EPA018LGAA12C000855	2	-	-	-	-
EPA018LGAA12C000638	2	EUB63160.1	1.95e-07	Murinoglobulin-2	-
EPA018LGAA12C000838	2	CDJ18319.1	4.40e-24	Hypothetical protein EgrG_001037900	-
EPA018LGAA12C000772	2	-	-	-	-
EPA018LGAA12S001747	1	-	-	-	-
EPA018LGAA12S004749	1	CDI70591.1	8.15e-42	Armet protein	33%
EPA018LGAA12S003348	1	CDJ11019.1	2.28e-10	Collagen alpha(iv) chain	56%
EPA018LGAA12S001839	1	-	-	-	-
EPA018LGAA12S002027	1	AFM74226.1	7.49e-16	Cysteine-rich with egf-like domains protein	34%
EPA018LGAA12S004089	1	CDJ21221.1	6.76e-51	Leucine rich repeat typical subtype	33%
EPA018LGAA12S003220	1	-	-	-	-
EPA018LGAA12S002557	1	CDJ24800.1	1.67e-54	Heat shock protein DnaJ N terminal	56%
EPA018LGAA12S003645	1	CDJ12970.1	1.81e-23	Type II collagen B	38%
EPA018LGAA12S000676	1	-	-	-	-
EPA018LGAA12S003769	1	-	-	-	-
EPA018LGAA12S004845	1	-	-	-	-
EPA018LGAA12S000743	1	-	-	-	-
EPA018LGAA12S000277	1	-	-	-	-
EPA018LGAA12S001291	1	CAJ00244.1	2.87e-11	TPA: endonuclease-reverse transcriptase	-
EPA018LGAA12S000397	1	AAM82156.1	1.03e-11	AF523312_1 oncosphere-specific antigen	42%
EPA018LGAA12S003589	1	XP_007441014.1	5.03e-28	PREDICTED: c-C motif chemokine 4-like	46%

### Transcriptome-wide comparison and parasitism

**Figure 3:**
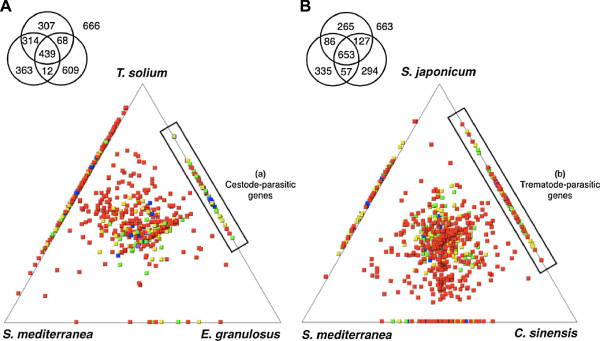
**Transcriptome-wide relative similarity between sparganum and other species.** Spargana contigs and singlets were searched against the whole transcriptome using TBLASTX score (a cut-off of ≥50). The Venn diagrams show the number of spargana sequences associated with each dataset. Global similarity comparison of cestoda **(A)** and trematoda **(B)** with a free-living flatworm. Square tiles indicate genes, with the squares colored by their highest TBLASTX score to each of the databases: red ≥300; yellow ≥200; green ≥150, blue ≥100 and purple <100.

To investigate the relative similarity between spargana and four parasitic flatworms and a free-living one, TBLASTX was performed against other organisms with publicly available ESTs and the degree of similarity was figuratively displayed using SimiTri program [31]. These included *Taenia solium* (30,587 ESTs) and *Echinococcus granulosus* (10,091 ESTs), *Clonorchis sinensis* (13,305 ESTs) [60] and *Schistosoma japonicum* (24,796 ESTs) and *Schmidtea mediterranea* (78,720 ESTs). Spargana (1,794 SpAEs) was more close to *T. solium* than *E. granulosus* (Figure 3A). This result showed the phylogenetic closeness within Eucestoda of class Cestoda. Evolutionary relationships of tapeworms represent a monophyletic group based on small (SSU) and large (LSU) subunit ribosomal DNA sequences and morphological characteristics [61]. *S. erinacei* (Cestoda, Pseudophyllidea) is sister group to *Taenia sp.* (Cestoda, Cyclophyllidea) while *E. granulosus* (Cestoda, Cyclophyllidea) forms a group with *Gyrocotyle rugosa* (Gyrocotylidea) [62]. When compared to both *C. sinensis* and *S. japonicum* (Trematoda, Digenea), SpAEs were scattered across two flukes’ transcriptomes (Figure 3B). Comparison of Pseudophyllidea with Digenea encompasses diversity across the parasitic Neodermata including Cestoda and Trematoda [63].

We identified 28 SpAEs, which were predicted to be helminth-parasitic genes in the intersection between cestode-parasitic genes (a) and trematode-parasitic genes (b) in the Figure 3 (Additional file 3: Table S2). These proteins in parasitic helminth were absent from the corresponding molecules in the free-living S. *mediterranea* (Turbellaria, outside of Neodermata) [64]. Of these, 9 showed sequence similarity neither to a gene/protein of known function nor to an identifiable protein domain. Due to the presence of these gene products only within parasitic helminths, and although their full characterization is needed, they may be good candidates for the development of potentially novel parasitic helminth drug targets. From the BLAST analyses, 537 SpAEs did not have any homologs in the analyzed species (Additional file 4: Table S3). These gene products can be explored as potential species-specific antigen candidates against sparganosis.

## Conclusions

This study is the first to analyze and characterize the transcriptome of *S. erinacei* spargana. This project provides an all-inclusive overview and preliminary analyses for genomic research on *S. erinacei* spargana and is a useful starting point for gene discovery, new drug development, novel antigen identification, and comparative analyses of genomes. In addition, this study will help facilitate whole genome sequencing and annotation.

## Electronic supplementary material

Additional file 1: Table S1.: Functional annotation of 1,794 SpAEs. This file contains BLASTX hits for each SpAE through BLAST2GO. (XLSX 255 KB)

Additional file 2: Figure S1.: Distribution of taxonomic groups of BLAST top hit species. (PDF 57 KB)

Additional file 3: Table S2.: SpAEs of *S. erinacei* spargana of potential genes associated with helminth parasitism. (XLS 105 KB)

Additional file 4: Table S3.: SpAEs for spargana-specific antigen candidates against sparganosis. (XLS 100 KB)
