# Supplementary figures and images for: Transcriptome sequencing and analysis of the zoonotic parasite Spirometra erinacei spargana (plerocercoids)
Source: Parasit Vectors. 2014 Aug 15;7:368. doi: 10.1186/1756-3305-7-368 (PMC4262225; doi:10.1186/1756-3305-7-368)

# Top-Hit species distribution

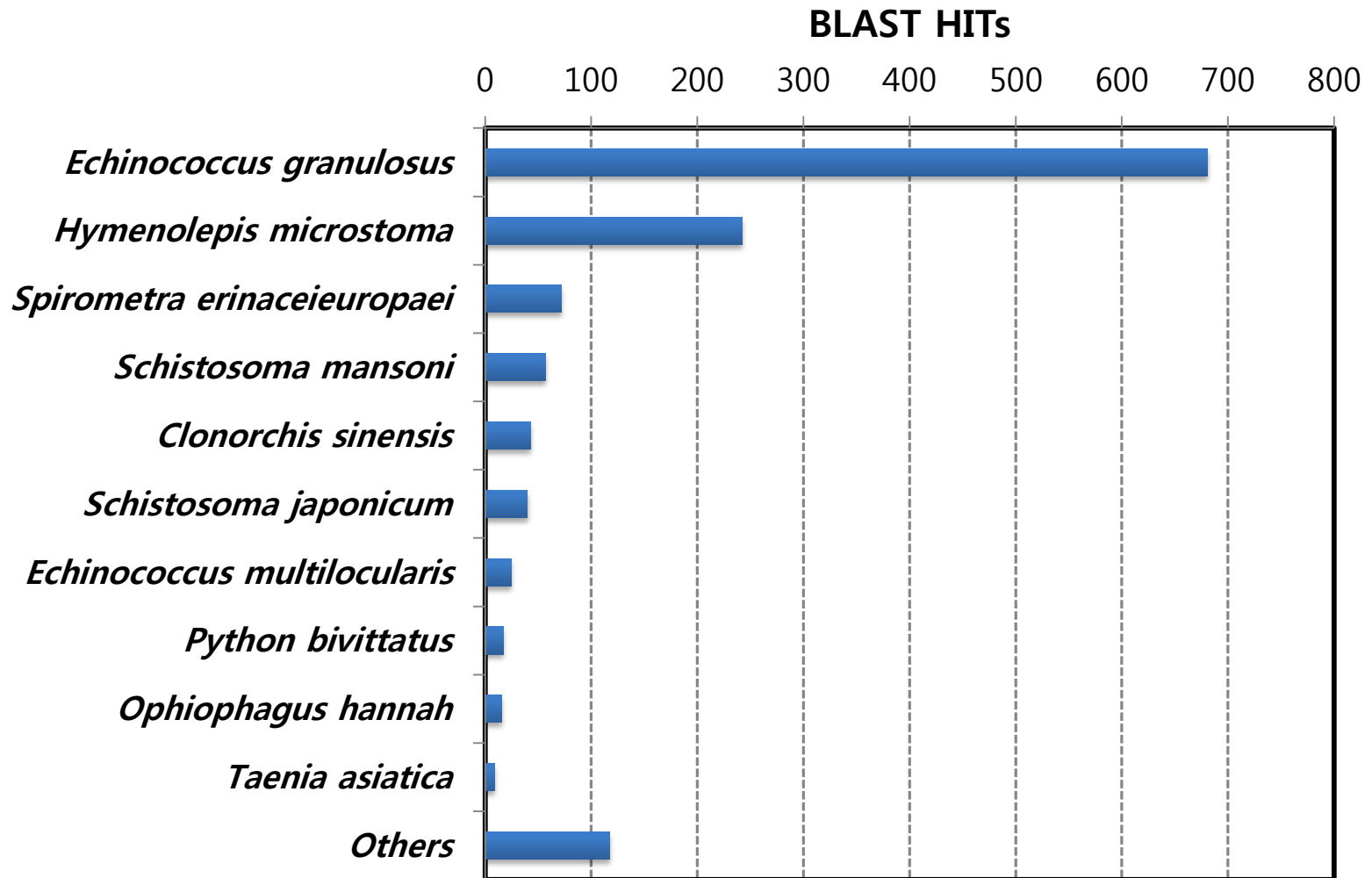

Supplement: Supplementary file 2 — Additional file 2: Figure S1.: Distribution of taxonomic groups of BLAST top hit species. (PDF 57 KB) [file 13071_2014_1634_MOESM2_ESM.pdf]
